# Expanding perigraft seroma after ascending aorta replacement

**DOI:** 10.1186/s13019-022-02018-y

**Published:** 2022-10-04

**Authors:** Shinji Kanemitsu, Shunsuke Sakamoto, Satoshi Teranishi, Toru Mizumoto

**Affiliations:** Department of Cardiovascular Surgery, Anjo Kosei Hospital, 28 Higashihirokute, Anjo-cho, Anjo, Aichi 446-8602 Japan

**Keywords:** Perigraft seroma, Dacron graft, Thoracic aorta

## Abstract

**Background:**

Perigraft seroma is a persistent and sterile fluid confined within a fibrous pseudomembrane surrounding a graft that develops after graft replacement. Development of perigraft seroma is an uncommon complication that occurs after the surgical repair of the thoracic aorta using woven polyester grafts. mechanism underlying perigraft seroma formation remains unclear.

**Case presentation:**

Herein, we describe the case of 77-year-old man who underwent repeat sternotomy for the treatment of large perigraft seroma 1 year after ascending aorta replacement for acute type A dissection. After removing a cloudy yellow fluid, we covered the prosthetic graft with fibrin glue and wrapped it with a new graft. Bacterial culture and laboratory examination of the fluid confirmed the final diagnosis of perigraft seroma, and there was no evidence of recurrence. The area in which fluid accumulated around the graft shrunk 1 year after surgery.

**Conclusions:**

The cause of a expanding perigraft after repair of the thoracic aorta remains unknown. Physicians should be aware that chronic expanding mediastinal seroma with Dacron grafts is one of the rare postoperative complications of thoracic aortic surgery. Applying fibrin glue to the graft surface might effectively prevent the recurrence of perigraft seroma.

**Supplementary Information:**

The online version contains supplementary material available at 10.1186/s13019-022-02018-y.

## Background

Perigraft seroma (PGS) is a persistent, sterile fluid collection that can develop after various vascular procedures. Development of PGS is relatively common in superficially placed graft (e.g., axillofemoral and femorofemoral bypasses), abdominal aortic aneurysm (AAA), and modified Blalock–Taussig shunts, whereas mediastinal PGSs occur less frequently. Moreover, PGS requiring repeat sternotomy after thoracic aortic surgery with a polyester graft is extremely rare. We present a case of PGS that developed after a polyester graft was used for ascending aorta replacement for acute type A dissecting aneurysm.

## Case presentation

**Fig. 1:**
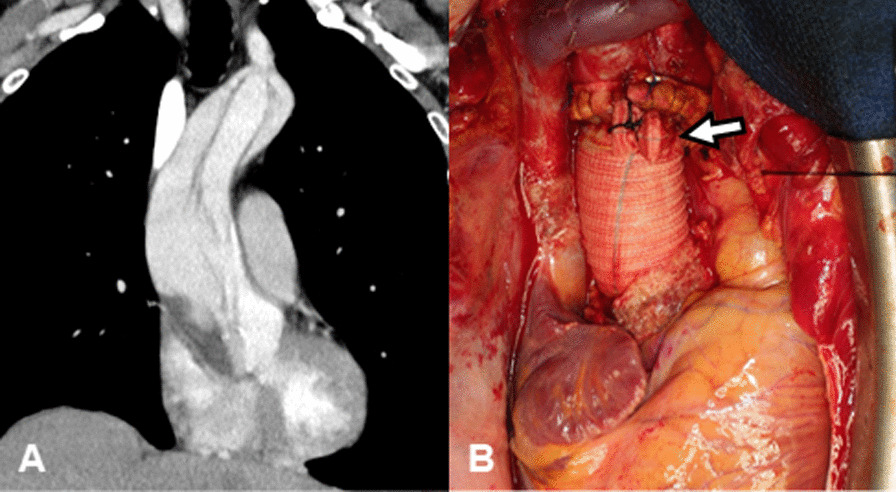
**A** Computed tomography angiography showing dissection of the ascending aorta. **B** Intraoperative photograph of the completed ascending aortic graft replacement. The side branch of the graft was ligated (arrow)

**Fig. 2:**
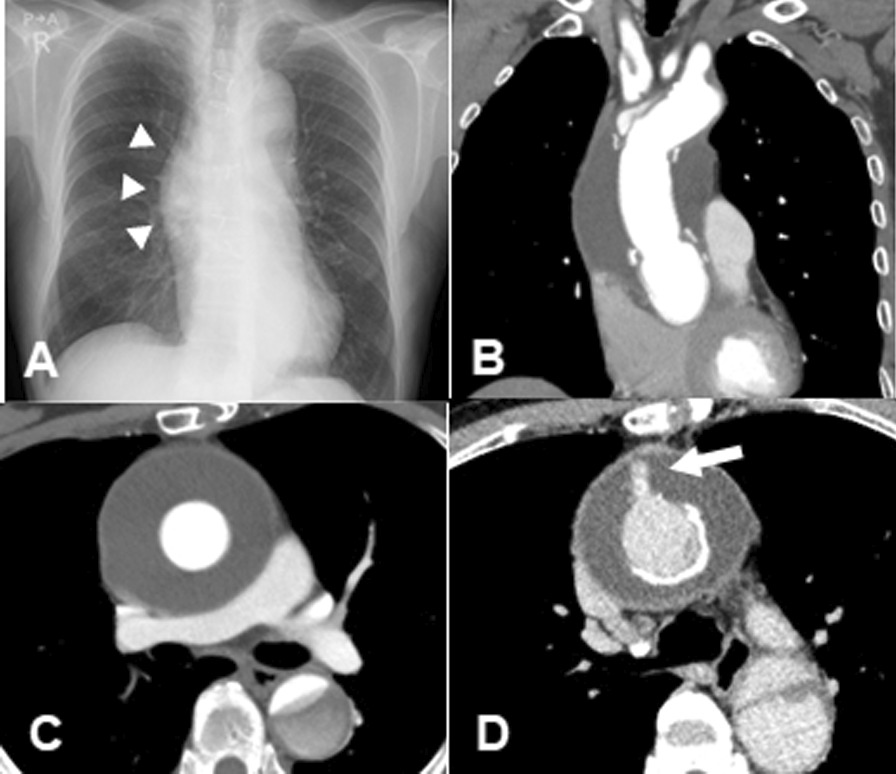
**A** Chest X-ray revealed the development of a right mediastinal eccentric convex abnormal contour (arrowhead). **B**, **C** Computed tomography 1 year after the first surgery showed the expanded large low-density area around the graft (70 × 74 mm). **D** A contrast effect in the side branch of the graft led to the suspicion of leakage from the stump of the side branch

**Fig. 3:**
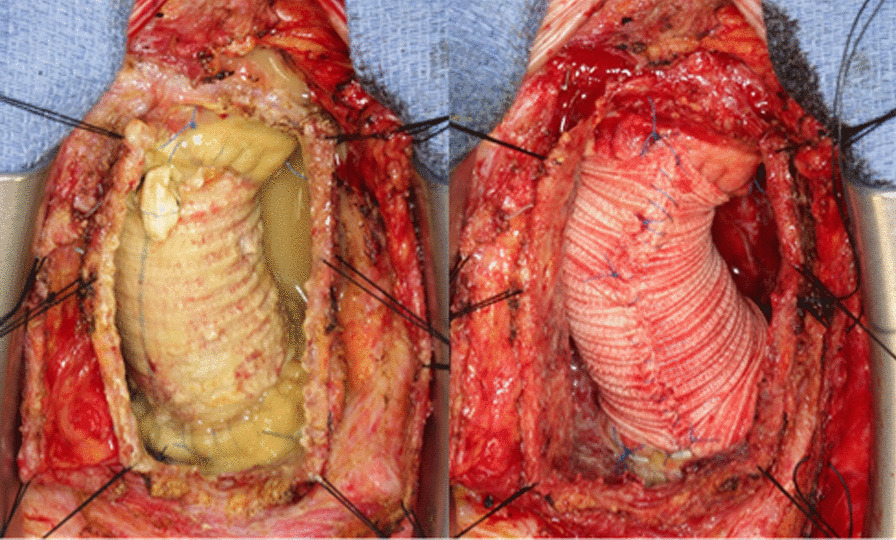
Intraoperative photograph. Cloudy yellow fluid around the graft was drained. The new graft was wrapped tightly after applying fibrin glue

The patient has provided permission to publish these features of his case, and the identity of the patient has been protected. A 77-year-old man underwent emergency ascending aorta replacement with a polyester woven graft (26-mm J-graft; Japan Lifeline, Tokyo, Japan) for Stanford type A aortic dissection (Fig. 1A, B). Postoperative computed tomography (CT) performed nonspecific effusion surrounding the graft. He was not administered any anticoagulants or antiplatelet drugs. 1 year after surgery, chest X-rays revealed the interval development of a right hilum overlay sign manifesting as an eccentric focal convex mediastinal contour abnormality (Fig. 2A). CT revealed a large low-density area within the abnormality, measuring 70 × 74 mm in diameter, which indicated circumferential perigraft fluid collection around the entire length of the graft in the ascending aorta (Fig. 2B, C). This fluid accumulation was 15 mm larger than the mass observed at postoperative 6 months. There was an absence of active contrast extravasation, inflammatory changes, bubbles, and wall enhancement. There was no evidence of prosthetic graft compression; however, the left pulmonary artery was compressed by the large mass. The average radiodensity of the mass was 20 HU, which suggested a seroma rather than a blood clot. CT with atrial contrast showed no evidence of a leak or pseudoaneurysm. However, the mass tended to expand, and consequently, compress the surrounding tissue. Although the patient was asymptomatic and had no remarkable inflammatory changes, we could not completely rule out the collapse of the anastomotic site, graft infection, and leakage from the remnant stump of the graft side branch (Fig. 2D). We therefore performed surgery to remove the massive amount of cloudy yellow fluid but found no bleeding site. We then applied fibrin glue to the surface of the graft to prevent serum leakage and tightly wrapped a new graft around it (Fig. 3, Additional file 1: Video 1). The fluid was negative for bacterial and fungal cultures. Laboratory test results for the fluid and blood were as follows: hemoglobin, 1.0 and 13.4 g/dl; total protein, 3.6 and 7.3 g/dl; albumin, 0.7 and 3.8 g/dl; lactate dehydrogenase, 2026 and 163 U/l; and triglycerides, 31 and 98 mg/dl, respectively. Histological examination of the wall of the mass revealed fibrous tissues and the infiltration of inflammatory cells. The results of these examinations confirmed the final diagnosis of perigraft seroma (PGS). There was no evidence of recurrence 1 year after surgery.

## Discussion and conclusions

Sundaram et al. reported the spectrum of CT findings and clinical outcomes among patients with thoracic aortic graft complications detected on CT [1]. CT-evident complications were identified in approximately 2.2% of the cases over a 7-year period, which suggests an extremely low complication rate. The most frequent type of CT-evident complication is the abnormal accumulation of low-attenuation material around the graft (51%), followed by collections of contrast material outside the graft (33%). PGS can be devastating as it can cause secondary graft infection, and PGS symptoms can vary depending on the site of formation. An asymptomatic mass [2], pain, acute limb ischemia secondary to graft limb compression, and respiratory distress have been reported [3].

Identifying PGS in the clinical setting is difficult, and the differential diagnoses include infection, pseudoaneurysm, postoperative hematoma, and lymphatic fluid collection. The accumulation of low-attenuation perigraft fluid is most often caused by infection, which must be ruled out. Bleeding because of anastomotic dehiscence without infection is a less frequent cause that is more frequently encountered in the early postoperative period and needs to be ruled out as well. In approximately 50% of the cases, the cause of low-attenuation perigraft fluid collection is not identified [1], and the patients remain asymptomatic. Kadakol et al. [3] defined PGS as a perigraft fluid collection present for > 3 months after surgery, with a diameter ≥ 3.0 cm and a radiodensity ≤ 25 HU. The present case met all three of these criteria. The mechanism underlying PGS formation remains unclear, but it is hypothesized to be the result of postoperative seroma and/or inflammatory edema developing because of an allergic reaction to the aortic graft material. Yamamoto et al. [4] suggested that collagen-impregnated vascular grafts contain contaminants with endotoxins and (1–3) b-d-glucan, which may cause a sterile inflammatory response around the graft. Whether the graft serves as a predisposing factor for PGS formation is controversial. Knitted Dacron grafts were most frequently used, followed by polytetrafluoroethylene [5]. Kadakol et al. [3] reported that diabetes, smoking, anticoagulation, bifurcated graft reconstruction, and left flank retroperitoneal approach were independent risk factors for the development of PGS after the open surgical repair of AAA.

Although the success rate of surgical intervention is unclear because of the rarity of PGS, several cases of successful surgical intervention have been reported. Ohtake et al. [6] reported the successful endovascular therapy for PGS of the descending aorta. Kadakol et al. [3] reported that 4 (20%) of 20 patients with PGS required intervention after the open surgical repair of AAAs, and graft replacement with another type of graft was performed in 2 (10%) patients. One of the treatment options is drainage; however, because of recurrence, endovascular repair or surgery might be necessary in select cases. In the present case, the diagnosis was confirmed using a combination of radiodensity on preoperative CT, bacterial culturing, and laboratory and histological examinations. The cause of a gradually expanding PGS after repair of the thoracic aorta remains unknown; however, we believe that applying fibrin glue to the graft surface and wrapping a new graft around it prevents the recurrence of fluid accumulation around the prosthetic graft. It has been reported that fibrin glue reduces the inflammatory process [7]. Higashi et al. [8] demonstrated that the new method, rubbing solution of fibrin glue with the finger, increased the resistance to pressure.

Physicians should be aware that chronic expanding mediastinal seroma with Dacron grafts is one of the rare postoperative complications of thoracic aortic surgery. Applying fibrin glue to the graft surface might effectively prevent the recurrence of PGS.

## Supplementary Information

**Additional file 1: Video 1**. Cloudy yellow fluid around the graft was drained. The new graft was wrapped tightly after applying fibrin glue.

## Data Availability

The data are not available for public access due to patient privacy concerns but are available from the corresponding author upon reasonable request.

## Abbreviations

AAA: Abdominal aortic aneurysms
CT: Computed tomography
PGS: Perigraft seroma
